# The Bacillus of Whooping-Cough

**Published:** 1888-04

**Authors:** 


### *THE BACILLUS OF WHOOPING-COUGH.*

"Dr. Afanasieff has succeeded in finding and cultivating what he believes to be the true bacillus of whooping cough. This microbe differs distinctly from all other bacteria which have been described. It is somewhat like Friedlander's pneumonia bacillus, but is shorter and thinner than the latter; besides, in gelatin it does not form nailshaped cultures, those which are produced having no hemispherical head. Its potato cultures, too, are quite different from those obtained from Friedlander's bacillus. Afanasieff's bacillus exhibits a remarkable degree of vitality, for jelly cultures that have become dry and have been kept for four months, appearing under the microscope to be more or less destroyed, are still capable of producing fresh cultures when fresh media are inoculated from the dried mass. Dr. Afanasieff's researches were chiefly made from the sputum of some of his own children, who were affected with whooping-cough. The mouth was well washed out with a permanganate solution, and the mucus coughed up after the next paroxysm or two examined. In this mucus, after staining with methyl-violet, and in the pus-corpuscles contained in it, the bacilli could be seen with a magnifying power of from 700 to 1,000 (Zeissl's eye-piece 3 or 4, one-twelfth oil immersion objective) as short rods, sometimes single, sometimes in twos, or even in short chains running in the direction of the mucus, sometimes again in small clusters. Their length was from 0.6 $\mu$ o 2.2 $\mu$. Of course, other bacteria were found. Pure cultures, however, were easily made on agar-agar meat, peptone jelly, potato, etc. Dogs and rabbits were inoculated with a fluid culture mixed with chloride of sodium solution, some by means of injection into the trachea, others by direct injections into the lungs. All the animals were seriously affected, and many of them died. The symptoms were somewhat similar to those of whooping-cough, including cough, dyspnœa, and redness of the eyes. Many of the cases were complicated by broncho-pneumonia. On examining the bodies of those animals which died, the mucous membrane of the air-passages was found much reddened, and coated with a tenacious, clear mucus, in which, as well as in the pneumonic patches in the lungs, the bacilli were found. Similar appearances and the same bacilli were observed in the bodies of children who had died from whooping cough. As to treatment, inhalations and sprays of various antiseptic drugs would appear to afford the most ground for hope in relieving and shortening this complaint. The author points out that the quinine, benzoin and other substances which have been used as applications to the nasal mucous membrane by Michael and others, under the idea of combating a reflex affection, are really, perhaps, beneficial, from their property of destroying bacilli.''

[Whether Afanasieff's bacillus has "come to stay" or not, there can be no question that pertussis is due to some specific germ, and that the proper treatment of the disease demands a recognition of the microbe. Elsewhere in the *Review* is a paper upon the use of the peroxide of hydrogen; the application of this remedy to the air passages in the treatment of whooping cough is referred to, and two cases cited. Of course, this is not enough to establish its value in this condition only so far as it goes.]

[In conjunction with the above, antipyrin, given four or five times in the twenty-four hours (a dose 3 to 4 grains to a child ten years old), the paroxysms were markedly diminished in frequency and severity, and the continuance of the disease abruptly shortened in spite of its occurrence being in the most unfavorable season—the severe weather of January and December. I was led to the administration of antipyrin by the fact that Prof. Germain See, of Paris, prescribed the drug successfully to a friend and patient of mine, who consulted him suffering with asthma. I have since observed in the *Medical Chronicle* the statement that Sonnenberg, in the *Deutsche Med. Woch.*, recommended it as the best remedy in whooping cough.]

"He has used it in seventy cases, and asserts it surpasses in efficacy and utility all other remedies. He gives one-seventh of a grain to very young children, and gradually increases the dose according to the age. To adults he gives fifteen grains. The medicine is administered three times daily, and sometimes once during the night. Children take it readily when dissolved in a little water and raspberry juice. The remedy should be continued throughout the attack, the cessation of it being accompanied by exacerbation of the cough. The average duration of cases thus treated is three to five weeks, and it rarely happens that more than six or seven paroxysms occur during twenty-four hours. Under antipyrin, the appetite and nutrition was found to improve. When Sonnenberg commenced this treatment at a late stage of this disease, he found that the paroxysms were at once reduced in violence, and that the expectoration was rendered more easy. In a few cases, the good effects of antipyrin were not noticed until a few days after its use had been commenced. Collapse was never noticed. Five out of seventy cases treated with antipyrin had complications; two suffered from pneumonia, and one of these died. The hygienic conditions of the patient should, of course, be attended to, but Sonnenberg says that though in the majority of his cases this was not possible, the curative influence of the drug was very definite."

*En passant* I desire to state that the pronouncement against the use of antipyrin in treating the diseases of children has not been borne out by my experience. I recognize the fact that the drug in certain conditions is more or less depressing, and needs to be given guardedly, but this will apply to all medicine. None should be given carelessly or by unskillful hands. To the mother possessed of a mania for dosing, the "inert infinitesimals are a great boon," and she should be advised to confine her manipulations to that particular field of harmlessness.]—*Weekly Med. Review.*

